# Limited value of serum neutrophil-to-lymphocyte ratio in the diagnosis of chronic periprosthetic joint infection

**DOI:** 10.1186/s10195-021-00599-3

**Published:** 2021-09-18

**Authors:** Yongyu Ye, Weishen Chen, Minghui Gu, Qiaoli Liu, Guoyan Xian, Baiqi Pan, Linli Zheng, Xiaoling Chen, Ziji Zhang, Puyi Sheng

**Affiliations:** Department of Orthopedics, The First Affiliated Hospital, Sun Yat-Sen University, 58 Zhongshan 2nd Road, Guangzhou, 510080 China

**Keywords:** Chronic periprosthetic joint infection, Neutrophil-to-lymphocyte ratio, Diagnosis

## Abstract

**Background:**

Diagnosing chronic periprosthetic joint infection (PJI) is challenging. No single biomarker can accurately recognize PJI preoperatively in a timely manner. Therefore, the aim of the present study was to investigate the usefulness of the serum neutrophil-to-lymphocyte ratio (NLR) in aiding the diagnosis of chronic PJI.

**Materials and methods:**

We retrospectively evaluated the medical records of 158 patients who had undergone revision arthroplasty (104 with aseptic mechanic failure and 54 with chronic PJI) from July 2011 to July 2020. Univariate analysis followed by multivariate logistic regression was applied to compare NLR, C-reactive protein (CRP), and erythrocyte sedimentation ratio (ESR) between the two groups. The receiver operating characteristic (ROC) curve was used to assess the diagnostic performance of NLR alone and in combination with CRP and ESR.

**Results:**

NLR, CRP, and ESR were significantly higher in patients with chronic PJI than in the aseptic revision group (*p* < 0.05). ROC curve analysis revealed that NLR had a sensitivity of 57.41% and a specificity of 77.88% with an optimal threshold of 2.56. The optimal threshold for CRP and ESR was 7.00 mg/L (sensitivity 62.50% and specificity 83.12%) and 43 mm/h (sensitivity 59.38% and specificity 80.52%), respectively. The combined diagnostic value of NLR with CRP and ESR was shown to have no additional diagnostic value in predicting chronic PJI.

**Conclusion:**

Compared with traditional inflammatory biomarkers (ESR and CRP), the value of serum NLR alone or combined with CRP and ESR for diagnosing chronic PJI is limited.

**Level of evidence:**

Level 3.

## Introduction

Although significant progress has been made in total joint arthroplasty (TJA), periprosthetic joint infection (PJI) remains one of the most disastrous postoperative complications. It leads to a lengthy hospital stay, creates a substantial economic burden, and increases morbidity and mortality rates [1–3]. It has been claimed that approximately 1–2.5% of orthopedic patients undergoing primary TJA will develop PJI [3–5]. The timely and accurate diagnosis of PJI continues to be a challenge. Despite the suggestion of several biomarkers, including C-reactive protein (CRP) and erythrocyte sedimentation rate (ESR), for the diagnosis of chronic PJI, as recommended by the Musculoskeletal Infection Society (MSIS), there are still no well-documented and reliable biomarkers that could help surgeons obtain the right and timely preoperative diagnosis [1, 6]. Both CRP and ESR could be within the normal range in some patients with PJI, particularly in those with chronic and low-virulence infections [7]. Additionally, the guideline requires the performance of an invasive procedure (joint aspiration and synovial fluid analysis/culture) or intraoperative exploration. Therefore, the requirement for easily accessible, noninvasive, and cost-effective biomarkers to assist with the timely and correct diagnosis of PJI is warranted.

The serum neutrophil-to-lymphocyte ratio (NLR), an easily accessible, cost-effective, and reproducible marker, is calculated from the complete blood count (CBC), which is recognized as a well-established biomarker of the systemic inflammatory response [8]. The NLR has been demonstrated to be a crucial biomarker for solid tumors and systemic rheumatic diseases, such as rheumatoid arthritis [9–11]. Recently, studies have shown that NLR was related to postoperative pain and surgical site infection in orthopedic surgery and might be a predictive biomarker for deep vein thrombosis after total knee arthroplasty [12–14]. Additionally, Golge reported that NLR might be useful in the diagnosis of PJI and that it increased diagnostic accuracy when combined with CRP and ESR [15]. Moreover, two studies have been carried out to demonstrate that NLR has a great ability to predict early PJI [16, 17]. However, to the best of our knowledge, no solid evidence was found regarding the diagnostic value of NLR in predicting chronic PJI.

Hence, the present study aimed to investigate the diagnostic performance of NLR in predicting chronic PJI in comparison with that of CRP and ESR in patients with total hip or knee arthroplasties and explore the complementary value of NLR in the diagnosis of chronic PJI when combined with CRP and ESR.

## Materials and methods

### Study design, selection criteria, and data review

Following approval by our institutional review board, we conducted a single-center, retrospective review of patients who had undergone revision total hip or knee arthroplasties from July 2011 to July 2020 in our hospital. Initially, a total of 222 revision patients were enrolled. Sixty-four patients were excluded from our study based on the following exclusion criteria: (1) patients with underlying inflammatory conditions, e.g., rheumatoid arthritis (*n* = 4) and ankylosing spondyloarthritis (*n* = 3); (2) patients experiencing other infectious conditions, such as tuberculosis (*n* = 3) and carbuncle (*n* = 2); (3) patients who had undergone revision surgery because of acute PJI (occurred < 3 months from the primary surgery, *n* = 1), dislocation (*n* = 16), periprosthetic fracture (most of them were due to violence, *n* = 5), breakage of prosthesis (*n* = 1), and surgical site infection (*n* = 10); (4) patients with severe dysfunction of kidney (*n* = 2); and (5) patients with lack of preoperatively recorded CRP (*n* = 9) or ESR (*n* = 8) values. Finally, the medical records of 104 patients with aseptic mechanic failure (aseptic loosening and wear, malalignment, instability, or other unexplained pain) and 54 patients with septic revision were analyzed in the study. The study protocol was performed in compliance with the Helsinki Declaration [18]. The requirement for informed consent was waived due to the retrospective design of our study. A detailed evaluation of medical records of all revision patients was carried out to review the demographic information and medical history. Baseline information, comorbidities, operative details, treatments, and complications were extracted from electronic records.

### Diagnostic criteria and protocol

To prevent infections after primary surgery, second-generation cephalosporins were frequently recommended intraoperatively and postoperatively within 24 h. In the event of redness, swelling, or any other evidence of infection in the wound or surgical site, antibiotics will be continued or upgraded to high-level antibiotics. The diagnosis of chronic PJI in our institution was defined by the MSIS criteria [1, 6]. After the comprehensive evaluation of patients, those for which clinical suspicion of PJI existed, who had significantly elevated serological infection-related biomarkers, or who had positive imaging findings were subjected to aspiration and subsequent bacterial culture. Moreover, analysis of the synovial fluid (color, clarity, Rivalta test [19], white blood cell count, polymorphonuclear differential, and Gram stain as needed) was also required. Preoperative antibiotics were not generally provided for PJI patients unless we were given specific instructions based on the findings of the bacterial culture. Additionally, cultures were obtained from different sites intraoperatively before the irrigation of iodine fluid. Recently, pathologic periprosthetic tissues were biopsied during surgery and sent for histologic analysis (43% of revised patients). Empirical second-generation cephalosporin was routinely initiated during and continued after revision surgery. Sensitive antibiotics would be further prescribed according to the sensitivity test of bacterial culture. All patients received prophylactic heparin or other antithrombotic therapy to prevent deep vein thrombosis within 24 h postoperatively and for continuous 28 days. Lastly, CBC and infection-related biomarkers were regularly reexamined to monitor their fluctuations.

### Laboratory evaluation

Routine blood samples, such as CBC, were drawn in the morning after patients had fasted for at least 8 h. In revision patients, CRP and ESR were also requested. Samples were sent to our hospital’s medical laboratory as soon as possible, in accordance with accepted standards and guidelines. Neutrophils and lymphocytes were extracted from CBC retrospectively. The NLR was calculated as the proportion of the absolute count of neutrophils to the absolute count of lymphocytes.

### Statistical analysis

Mean and standard deviation (SD) were used to describe the quantitative data, while the number and percentage were calculated for categorical variables. An independent *t*-test was performed to detect differences in age between groups. The categorical variables of demographic features were calculated with the use of a *χ*^2^ test. To evaluate the differences of each serologic biomarker between aseptic and septic groups, we used a Mann–Whitney *U* test for comparisons. To decrease the effect of confounding factors, the adjusted *p*-value was assessed by multivariate logistic regression (forward likelihood ratio method) regarding age, sex, operative joint, diabetes mellitus, and hypertension. The above analyses were performed using the Statistical Package for the Social Sciences (SPSS) (version 22, IBM Corporation, Armonk, NY, USA). MedCalc software (version 19.0.7, Ostend, Belgium) was employed to analyze the receiver operating characteristic (ROC) curves. Area under the curve (AUC), sensitivity, specificity, and other related parameters were used to investigate the diagnostic performance of NLR, CRP, ESR, and combination of biomarkers. The Youden index was applied to determine the optimal threshold for the diagnosis of PJI. Variables with *p* < 0.05 were considered statistically significant.

## Results

### Demographic features of patients undergoing revision surgery

The demographic features of both groups are presented in Table 1. Among patients with PJI, the average age was 64.5 years, with 31.5% being male. The baseline characteristics of age, sex ratio, and status of diabetes mellitus between the two groups did not exhibit significant differences. In the aseptic revision group, the percentage of patients with hip problems was significantly higher than in the PJI group (81.7% versus 50.0%, *p* < 0.001). Additionally, the two groups differed regarding the status of blood pressure, with the PJI group suffering more from hypertension (44.4% versus 21.2%, *p* = 0.002).

**Table 1:** Demographic features of patients who underwent revision surgery

Group and variable	Aseptic revision	PJI	*p*-Value
No. of patients	104	54	
Age^a^	63.40 ± 12.70	64.48 ± 10.83	0.596^c^
Sex^b^			0.453^d^
Female	65 (62.5%)	37 (68.5%)	
Male	39 (37.5%)	17 (31.5%)	
Joint^b^			< 0.001^d^
Hip	85 (81.7%)	27 (50.0%)	
Knee	19 (18.3%)	27 (50.0%)	
Diabetes mellitus^b^			0.557 ^d^
Diabetic	12 (11.5%)	8 (14.8%)	
Nondiabetic	92 (88.5%)	46 (85.2%)	
Blood pressure^b^			0.002^d^
Hypertension	22 (21.2%)	24 (44.4%)	
Nonhypertension	82 (78.8%)	30 (55.6%)	

*PJI* periprosthetic joint infection

^a^Data are presented as the mean ± standard deviation

^b^Data are presented as the number (percentage) of patients

^c^*p*-Value was calculated by the independent *t*-test

^d^*p*-Value was calculated by the *χ*^2^ test. *p* < 0.05 indicates a significant difference between groups

### Comparisons of serum biomarkers between patients in aseptic and septic groups

**Fig. 1:**
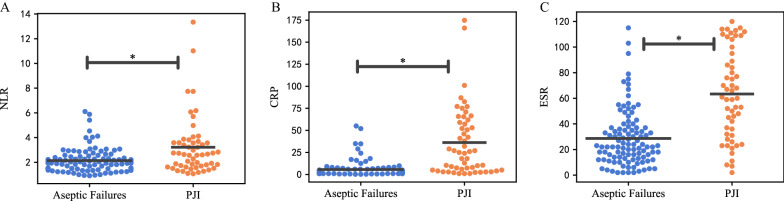
Comparisons of NLR, CRP, and ESR between aseptic mechanic failure and PJI patients. **a** NLR, **b** CRP, **c** ESR. The asterisks indicate a significant difference between groups. The *p*-values were calculated by the Mann–Whitney *U* test. *p* < 0.05 indicates a significant difference between groups. *NLR* neutrophil-to-lymphocyte ratio, *CRP* C-reactive protein, *ESR* erythrocyte sedimentation rate. The black line represents the mean value of each group

For all three biomarkers (NLR, CRP, and ESR), significant differences were noted between the aseptic and septic groups, as shown in Table 2 and Fig. 1. The NLR for patients with previous diagnosis of chronic PJI was 3.22 compared with 2.13 among patients in the aseptic revision group (*p* = 0.001, adjusted *p* = 0.008). The mean value of CRP in the PJI group was 36.25 mg/L, which was significantly higher than that of the aseptic revision group with a mean value of 5.57 mg/L (*p* and adjusted *p* < 0.001). Similarly, the level of ESR in patients with PJI was significantly different in comparison with that of the aseptic revision group (63.35 versus 28.66 mm/h, *p* and adjusted *p* < 0.001).

**Table 2:** Comparison of biomarkers between the aseptic and septic revision

Biomarker	Aseptic revision^a^	PJI^a^	*p*-Value	Adjusted *p*-Value
NLR	2.13 ± 0.97	3.22 ± 2.37	0.001	0.008
CRP (mg/L)	5.57 ± 9.27	36.25 ± 38.79	< 0.001	< 0.001
ESR (mm/h)	28.66 ± 22.17	63.35 ± 34.81	< 0.001	< 0.001

^a^Data are presented as the mean ± standard deviation. *p*-Value was calculated by the Mann–Whitney *U* test. Adjusted *p*-value was assessed by the multivariate logistic regression (forward likelihood ratio method) regarding age, sex, joint, diabetes mellitus, and hypertension. *p* < 0.05 indicates a significant difference between groups

*PJI* periprosthetic joint infection, *NLR* neutrophil-to-lymphocyte ratio, *CRP* C-reactive protein, *ESR* erythrocyte sedimentation rate

### The diagnostic performance of NLR, CRP, and ESR in detecting chronic PJI

**Fig. 2:**
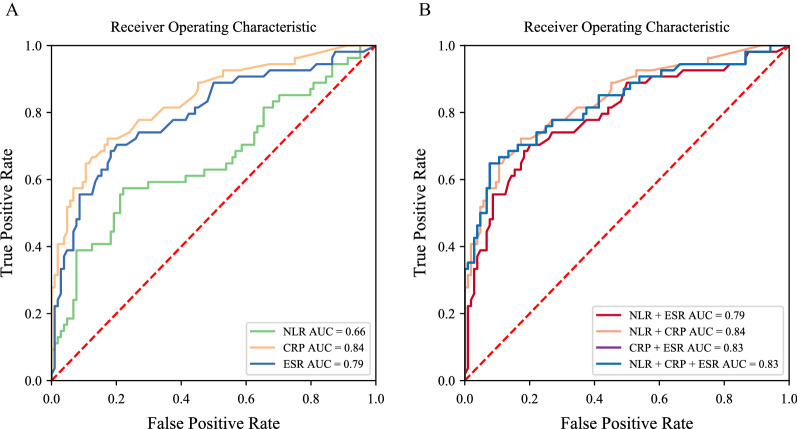
ROC curve of NLR, CRP, ESR, and combination to predict PJI. **a** The diagnostic performance of each biomarker alone. **b** The diagnostic performance of the combination of biomarkers. *NLR* neutrophil-to-lymphocyte ratio, *CRP* C-reactive protein, *ESR* erythrocyte sedimentation rate, *AUC* area under the curve, *PJI* periprosthetic joint infection

Consecutively, ROC curves were used to depict the discriminatory performance of NLR, CRP, and ESR between the aseptic mechanic failure group and the chronic PJI group. The respective AUC values for NLR, CRP, and ESR were demonstrated to be 0.66 [95% confidence interval (CI) 0.58–0.73, *p* = 0.001], 0.84 (95% CI 0.77–0.89, *p* < 0.001), and 0.79 (95% CI 0.72–0.85, *p* < 0.001). The sensitivity and specificity of NLR in detecting PJI were 57.41% and 77.88%, with an optimal threshold > 2.56. The optimal threshold with regard to CRP and ESR was evaluated to be 7.00 mg/L (sensitivity 72.22% and specificity 82.69%) and 43.00 mm/h (sensitivity 68.52% and specificity 81.73%), respectively. The diagnostic performance of NLR was therefore shown to be inferior to that of CRP and ESR. Additionally, we further evaluated the combined diagnostic value of NLR, CRP, and ESR for predicting chronic PJI. The resulting AUC for the combination of NLR with CRP and ESR was shown to have no additional diagnostic value. On the other hand, as opposed to using CRP and ESR alone, the specificity and diagnostic performance of CRP in conjunction with ESR (92.31 versus 82.69%, 81.73%) were significantly improved, enhancing the ability to detect PJI (Table 3; Fig. 2).

**Table 3:** Performance of serum NLR, CRP, ESR, and combination in the diagnosis of PJI

	NLR	CRP	ESR	NLR + CRP	NLR + ESR	CRP + ESR	NLR + CRP + ESR
AUC	0.66	0.84	0.79	0.84	0.79	0.83	0.83
95% CI	0.58–0.73	0.77–0.89	0.72–0.85	0.77–0.89	0.72–0.85	0.76–0.88	0.76–0.88
*p*-Value	0.001	< 0.001	< 0.001	< 0.001	< 0.001	< 0.001	< 0.001
Threshold	2.56	7.00	43.00	–	–	–	–
Sensitivity (%)	57.41	72.22	68.52	72.22	68.52	64.81	64.81
Specificity (%)	77.88	82.69	81.73	82.69	81.73	92.31	92.31
PPV (%)	57.4	68.4	66.1	68.4	66.1	81.4	81.4
NPV (%)	77.9	85.1	83.3	85.1	83.3	83.5	83.5
+LR	2.60	4.17	3.75	4.17	3.75	8.43	8.43
−LR	0.55	0.34	0.39	0.34	0.39	0.38	0.38
Accuracy (%)	70.88	79.11	77.22	79.11	77.22	82.91	82.91
DOR	4.75	12.42	9.74	12.42	9.74	22.11	22.11

*PJI* periprosthetic joint infection, *NLR* neutrophil-to-lymphocyte ratio, *CRP* C-reactive protein, *ESR* erythrocyte sedimentation rate, *AUC* area under the curve, *CI* confidence interval, *PPV* positive predictive value, *NPV* negative predictive value, * +LR* positive likelihood ratio, *−LR* negative likelihood ratio, *DOR* diagnostic odds ratio

## Discussion

The association between preoperative levels of NLR and chronic PJI is yet to be elucidated. In the present study, we investigated the diagnostic performance of the cost-effective and easily accessible NLR biomarker in facilitating the diagnosis of chronic PJI. We found that the level of NLR was significantly higher in the PJI group than in the aseptic mechanic failure group. However, the diagnostic value of NLR alone was demonstrated to not be a superior biomarker to those of CRP and ESR in diagnosing chronic PJI. Moreover, when we combined NLR with CRP and ESR, this combination was shown to have no additional diagnostic value. Nonetheless, the combination of CRP and ESR enhanced specificity and diagnostic performance, and thus improved the ability to detect chronic PJI.

NLR is calculated directly from the commonly ordered and readily available count of neutrophils and lymphocytes in CBC, making it easily accessible and cost effective. More importantly, NLR has been recognized as a biomarker of the systemic inflammatory response [8]. As such, NLR has been reported to reflect a heightened inflammatory reaction and has gained increasing attention as a prognostic biomarker in many conditions, such as solid tumors, inflammatory diseases, and postoperative infection [10, 11, 14]. A previous study reported that NLR was significantly higher in patients with polymyalgia rheumatica and was shown to be associated with disease activity and specific clinical features [10]. Inose et al. retrospectively investigated the association of NLR with surgical site infection in patients who underwent spinal instrumentation surgery. They found that the level of NLR postoperatively was significantly related to surgical site infection with a cutoff of 3.87 [14]. Regarding the role of NLR in TJA, Yombi et al. investigated the distribution and fluctuation of NLR compared with that of CRP in patients who underwent total knee arthroplasty and found that NLR had a faster normalization and was more stable than CRP, demonstrating that NLR might be utilized as a potential biomarker to monitor postoperative inflammation or infection [20]. In terms of the diagnostic value of NLR in predicting early PJI, Zhao et al. and Yu et al. both revealed that NLR was sensitive and has a potential ability to diagnose early PJI, even superior to CRP [16, 17]. However, for chronic PJI, although the level of NLR was significantly higher in patients with PJI in comparison with aseptic patients in our study, the alone or combined diagnostic value of NLR in predicting chronic PJI was limited. Our present study addressed that NLR had poor diagnostic performance with a sensitivity of 57.41% and specificity of 77.88%. In comparison, the study conducted by Golge [15] revealed that the sensitivity and specificity of NLR in detecting PJI were 90% and 72%, respectively, that is, the sensitivity was much higher than that of our study. However, the study involved patients with either PJI or primary total knee arthroplasty, with the primary arthroplasty patients being considered as the control group, which would have a great impact on the diagnostic performance of NLR because of the larger heterogeneity between the two groups. As the selection of patients is crucial for exploring the diagnostic value of biomarkers, patients should be generalized under consistent conditions to decrease the confounding effect [21]. Hence, future studies might need to clarify the diagnostic value of serum NLR in predicting PJI, especially for the chronic condition.

Many studies have delineated the value and established an optimal threshold of CRP and ESR in diagnosing chronic PJI. Both CRP and ESR, which are considered as specific indicators of infection, were shown to be highly elevated in the PJI group and to gradually decline postoperatively [22, 23]. The sensitivity of CRP for diagnosing PJI has been reported to range from 74% to 94%, whereas its specificity ranged from 20% to 100%, with different predictive cutoffs [22–26]. On the other hand, the sensitivity and specificity of ESR were reported to vary from 42% to 94%, and 33% to 90%, respectively [23–25, 27, 28]. In our study, the diagnostic performance of CRP and ESR in predicting PJI was consistent with previous studies, showing high effectiveness for diagnosing PJI. Additionally, when CRP combined with ESR in predicting PJI, the specificity and diagnostic performance were significantly improved, which was in line with previous studies demonstrating that combinations of biomarkers have been addressed to enhance diagnostic performance [22, 28–30]. Abdelbary et al. conducted a systematic review to assess the combined diagnostic performance of various serum, synovial, and tissue-based tests for PJI. They demonstrated that the combination of CRP and ESR enhanced the diagnostic performance dramatically in detecting PJI [29]. Paziuk et al. demonstrated that the ratio of the platelet count to mean platelet volume was inferior to CRP and ESR when employed as a single index. However, when the ratio was used in combination with CRP and ESR, a statistically significant increase in the diagnostic performance of the combination of biomarkers in predicting PJI was observed [28]. Qin et al. revealed that the combination of D-dimer and CRP improved the diagnostic performance in predicting PJI [30]. Therefore, CRP and ESR, as traditional inflammatory biomarkers, using alone or combined, exhibited promising value for diagnosing chronic PJI.

Our study has several limitations that should be considered when interpreting the results. First, this was a retrospective study and therefore might have introduced selection bias. Second, not all confounding variables were taken into account in the study. Third, the study only included patients who needed revision arthroplasty. As a result, it is possible that patients with asymptomatic infections or mild clinical manifestations were left out of the study. Finally, this was a single-center study with a small number of patients, particularly in the chronic PJI group. Recruiting more patients from multiple centers in a prospective study would allow for better representation.

## Conclusion

Our study revealed that serum NLR was significantly higher in patients with chronic PJI than in those with aseptic mechanical failure. Nevertheless, in comparison with traditional inflammatory markers, the alone or combined value of serum NLR for diagnosing chronic PJI is limited. More large-scale and prospective studies might be needed to further elucidate the value of NLR in the diagnosis of chronic PJI.

## Data Availability

The datasets used during the current study are available from the corresponding author on reasonable request.

## Abbreviations

TJA: Total joint arthroplasty
PJI: Periprosthetic joint infection
NLR: Neutrophil-to-lymphocyte ratio
CBC: Complete blood count
CRP: C-reactive protein
ESR: Erythrocyte sedimentation ratio
ROC: Receiver operating characteristic
SD: Standard deviation
AUC: Area under the curve
CI: Confidence interval
PPV: Positive predictive value
NPV: Negative predictive value
+LR: Positive likelihood ratio
−LR: Negative likelihood ratio
DOR: Diagnostic odds ratio

## References

[1] Parvizi J, Tan TL, Goswami K, Higuera C, Della Valle C, Chen AF, Shohat N (2018). The 2018 definition of periprosthetic hip and knee infection: an evidence-based and validated criteria. J Arthroplasty.

[2] Gwam CU, Mistry JB, Mohamed NS, Thomas M, Bigart KC, Mont MA, Delanois RE (2017). Current epidemiology of revision total hip arthroplasty in the United States: national inpatient sample 2009 to 2013. J Arthroplasty.

[3] Kurtz SM, Lau E, Watson H, Schmier JK, Parvizi J (2012). Economic burden of periprosthetic joint infection in the United States. J Arthroplasty.

[4] Jamsen E, Varonen M, Huhtala H, Lehto MU, Lumio J, Konttinen YT, Moilanen T (2010). Incidence of prosthetic joint infections after primary knee arthroplasty. J Arthroplasty.

[5] Huotari K, Peltola M, Jamsen E (2015). The incidence of late prosthetic joint infections: a registry-based study of 112,708 primary hip and knee replacements. Acta Orthop.

[6] Amanatullah D, Dennis D, Oltra EG, Marcelino Gomes LS, Goodman SB, Hamlin B, Hansen E, Hashemi-Nejad A, Holst DC, Komnos G, Koutalos A, Malizos K, Martinez Pastor JC, McPherson E, Meermans G, Mooney JA, Mortazavi J, Parsa A, Pécora JR, Pereira GA, Martos MS, Shohat N, Shope AJ, Zullo SS (2019). Hip and knee section, diagnosis, definitions: Proceedings of International Consensus on Orthopedic Infections. J Arthroplasty.

[7] Perez-Prieto D, Portillo ME, Puig-Verdie L, Alier A, Martinez S, Sorli L, Horcajada JP, Monllau JC (2017). C-reactive protein may misdiagnose prosthetic joint infections, particularly chronic and low-grade infections. Int Orthop.

[8] Mozos I, Malainer C, Horbanczuk J, Gug C, Stoian D, Luca CT, Atanasov AG (2017). Inflammatory markers for arterial stiffness in cardiovascular diseases. Front Immunol.

[9] Dolan RD, McSorley ST, Park JH, Watt DG, Roxburgh CS, Horgan PG, McMillan DC (2018). The prognostic value of systemic inflammation in patients undergoing surgery for colon cancer: comparison of composite ratios and cumulative scores. Br J Cancer.

[10] Jung JY, Lee E, Suh CH, Kim HA (2019). Neutrophil-to-lymphocyte ratio and platelet-to-lymphocyte ratio are associated with disease activity in polymyalgia rheumatica. J Clin Lab Anal.

[11] Templeton AJ, Knox JJ, Lin X, Simantov R, Xie W, Lawrence N, Broom R, Fay AP, Rini B, Donskov F, Bjarnason GA, Smoragiewicz M, Kollmannsberger C, Kanesvaran R, Alimohamed N, Hermanns T, Wells JC, Amir E, Choueiri TK, Heng DY (2016). Change in neutrophil-to-lymphocyte ratio in response to targeted therapy for metastatic renal cell carcinoma as a prognosticator and biomarker of efficacy. Eur Urol.

[12] Canbolat N, Buget MI, Sivrikoz N, Altun D, Kucukay S (2019). The relationship between neutrophil to lymphocyte ratio and postoperative pain in total knee and hip arthroplasty. Rev Bras Anestesiol.

[13] Barker T, Rogers VE, Henriksen VT, Brown KB, Trawick RH, Momberger NG, Lynn Rasmussen G (2015). Is there a link between the neutrophil-to-lymphocyte ratio and venous thromboembolic events after knee arthroplasty? A pilot study. J Orthop Traumatol.

[14] Inose H, Kobayashi Y, Yuasa M, Hirai T, Yoshii T, Okawa A (2019). Procalcitonin and neutrophil lymphocyte ratio after spinal instrumentation surgery. Spine (Phila Pa 1976).

[15] Golge UH, Kaymaz B, Pazarci O, Kilinc S, Oztemur Z, Bulut O (2016). Neutrophil to lymphocyte ratio may be a diagnostic marker for prosthetic joint infection. J Clin Anal Med.

[16] Zhao G, Chen J, Wang J, Wang S, Xia J, Wei Y, Wu J, Huang G, Chen F, Shi J, Lyu J, Liu C, Huang X (2020). Predictive values of the postoperative neutrophil-to-lymphocyte ratio, platelet-to-lymphocyte ratio, and lymphocyte-to-monocyte ratio for the diagnosis of early periprosthetic joint infections: a preliminary study. J Orthop Surg Res.

[17] Yu BZ, Fu J, Chai W, Hao LB, Chen JY (2020). Neutrophil to lymphocyte ratio as a predictor for diagnosis of early periprosthetic joint infection. BMC Musculoskelet Disord.

[18] World Medical Association (2013). World Medical Association Declaration of Helsinki: ethical principles for medical research involving human subjects. JAMA.

[19] Berti-Bock G, Vial F, Premuda L, Rulliere R (1979). Exudates, transudates and the Rivalta reaction (1895): current status and historical premises. Minerva Med.

[20] Yombi JC, Schwab PE, Thienpont E (2016). Neutrophil-to-lymphocyte ratio (NLR) distribution shows a better kinetic pattern than C-reactive protein distribution for the follow-up of early inflammation after total knee arthroplasty. Knee Surg Sports Traumatol Arthrosc.

[21] Bernard L, Lubbeke A, Stern R, Bru JP, Feron JM, Peyramond D, Denormandie P, Arvieux C, Chirouze C, Perronne C, Hoffmeyer P, Groupe D'Etude Sur LO (2004). Value of preoperative investigations in diagnosing prosthetic joint infection: retrospective cohort study and literature review. Scand J Infect Dis.

[22] Glehr M, Friesenbichler J, Hofmann G, Bernhardt GA, Zacherl M, Avian A, Windhager R, Leithner A (2013). Novel biomarkers to detect infection in revision hip and knee arthroplasties. Clin Orthop Relat Res.

[23] Xiong L, Li S, Dai M (2019). Comparison of D-dimer with CRP and ESR for diagnosis of periprosthetic joint infection. J Orthop Surg Res.

[24] Xu H, Xie J, Huang Q, Lei Y, Zhang S, Pei F (2019). Plasma fibrin degradation product and D-dimer are of limited value for diagnosing periprosthetic joint infection. J Arthroplasty.

[25] Saleh A, George J, Faour M, Klika AK, Higuera CA (2018). Serum biomarkers in periprosthetic joint infections. Bone Joint Res.

[26] Shahi A, Kheir MM, Tarabichi M, Hosseinzadeh HRS, Tan TL, Parvizi J (2017). Serum D-dimer test is promising for the diagnosis of periprosthetic joint infection and timing of reimplantation. J Bone Joint Surg Am.

[27] Ghanem E, Antoci V, Pulido L, Joshi A, Hozack W, Parvizi J (2009). The use of receiver operating characteristics analysis in determining erythrocyte sedimentation rate and C-reactive protein levels in diagnosing periprosthetic infection prior to revision total hip arthroplasty. Int J Infect Dis.

[28] Paziuk T, Rondon AJ, Goswami K, Tan TL, Parvizi J (2019). A novel adjunct indicator of periprosthetic joint infection: platelet count and mean platelet volume. J Arthroplasty.

[29] Abdelbary H, Cheng W, Ahmadzai N, Carli AV, Shea BJ, Hutton B, Fergusson DA, Beaule PE (2020). Combination tests in the diagnosis of chronic periprosthetic joint infection: Systematic review and development of a stepwise clinical decision-making tool. J Bone Joint Surg Am.

[30] Qin L, Li F, Gong X, Wang J, Huang W, Hu N (2020). Combined measurement of D-dimer and C-reactive protein levels: highly accurate for diagnosing chronic periprosthetic joint infection. J Arthroplasty.

